# O075: Successful implementation of the World Health Organization hand hygiene improvement strategy in a teaching hospital, China

**DOI:** 10.1186/2047-2994-2-S1-O75

**Published:** 2013-06-20

**Authors:** F Qiao, Z Zong, W Yin, W Huang, H Zhuang

**Affiliations:** 1 Infection Control Department, West China Hospital, Sichuan University, Chengdu, China

## Introduction

Although hand hygiene is the most effective measure for preventing health care –associated infections, overall compliance of hand hygiene is poor in developing countries, and it is hard to promote HH by using WHO multi-strategy.

## Objectives

To assess the feasibility and effectiveness of the World Health Organization hand hygiene improvement strategy in a 4300 beds hospital, a resource-poor area in China.

## Methods

A multi-prong approach was used in designing the hand hygiene program. The intervention consisted of rebuild hand-sinks; introducing a locally produced, self-branded alcohol-based handrub(ABHR); use the liquid soap take the place of solid soap; training and educating staff, students and patients through infection control week and World Hand Hygiene Day; monitoring hand hygiene compliance and the usage of ABHR and liquid soap, providing performance feedback; posting reminders in the workplace; and promoting an institutional safety climate according to the WHO multimodal hand hygiene improvement strategy.

## Results

All the hand-sinks were rebuild and hand-actuated taps were replaced. One self-branded ABHR has been produced with the cooperation of local manufacturers using the hand rub formulation provided by WHO. At follow-up, the usage of ABHR increased from 2.17(2009) to 12.28(2012) L per 1000 patient-days. And the compliance of hand hygiene increased from 45.9%(in Jan 2012) to 67.9% (in Dec. 2012). Improvement was observed across all professional categories. Healthcare-associated MRSA infection were reduced from 0.21( in January 2012) to 0.06(in Dec 2012) per 1000 patient-days.

## Conclusion

Multimodal hand hygiene improvement strategy is feasible and effective in a big teaching hospital in developing country. Leadership’s support of the program and the active participation of staff is the key factor in helping to make the program a true success. The WHO multimodal inventions work in improving compliance and reducing healthcare associated infections.

## Disclosure of interest

None declared.

